# Helicases FANCJ, RTEL1 and BLM Act on Guanine Quadruplex DNA *in Vivo*

**DOI:** 10.3390/genes10110870

**Published:** 2019-10-31

**Authors:** Peter Lansdorp, Niek van Wietmarschen

**Affiliations:** 1 Terry Fox Laboratory, British Columbia Cancer Agency, Vancouver, BC V5Z 1L3, Canada; 2 Department of Medical Genetics, University of British Columbia, Vancouver, BC V6H 3N1, Canada; 3 European Research Institute for the Biology of Ageing, University of Groningen, 9713 AV Groningen, The Netherlands; niek.vanwietmarschen@nih.gov; 4 Laboratory of Genome Integrity, National Cancer Institute, NIH, Bethesda, MD 20892, USA

**Keywords:** Guanine quadruplex (G4) DNA structures, G4 helicases, DOG-1, FANCJ, RTEL1, BLM, sister chromatid exchange events (SCEs), genomic mapping of SCEs, molecular phenotype, single cell Strand-seq

## Abstract

Guanine quadruplex (G4) structures are among the most stable secondary DNA structures that can form in vitro, and evidence for their existence in vivo has been steadily accumulating. Originally described mainly for their deleterious effects on genome stability, more recent research has focused on (potential) functions of G4 structures in telomere maintenance, gene expression, and other cellular processes. The combined research on G4 structures has revealed that properly regulating G4 DNA structures in cells is important to prevent genome instability and disruption of normal cell function. In this short review we provide some background and historical context of our work resulting in the identification of FANCJ, RTEL1 and BLM as helicases that act on G4 structures in vivo. Taken together these studies highlight important roles of different G4 DNA structures and specific G4 helicases at selected genomic locations and telomeres in regulating gene expression and maintaining genome stability.

## 1. G-Quadruplex Structures and G-Quadruplex Helicases

DNA molecules are capable of adopting a wide range of secondary structures besides the canonical B-DNA duplex form. Most secondary structures form when B-DNA is unwound during transcription or replication. Depending on the sequence context, single stranded DNA (ssDNA) can interact with itself or other DNA strands to form non B-DNA structures ranging from “simple” hairpins and cruciforms to more complicated structures such as guanine- quadruplex (G4) DNA. While many secondary structures have functions in e.g., regulating transcriptional activity, (nearly) all of them can form a barrier for progression of replication forks and must be resolved during DNA replication. To deal with the wide range of secondary structures that can form, it appears that all branches of life have evolved divergent repertoires of DNA helicases precisely for this role. The human genome encodes hundreds of helicases, many of which appear to have non-redundant functions. Most helicases are able to unwind different forms of DNA structures, at least in vitro, but display higher affinity for some structures than others. It seems safe to assume that many of such helicases have evolved specifically to unwind one or more different secondary DNA structures.

**Figure 1:**
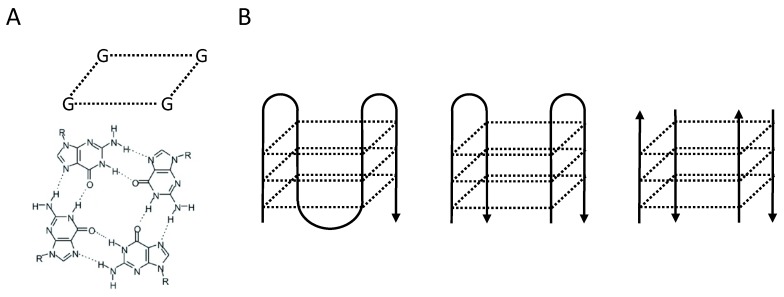
G-quadruplex structures form at G-rich DNA motifs. (**A**) Representation of a G-quartet formed by four guanines residues and stabilized by Hoogsteen base pairing. (**B**) Multiple G-quartets stack into G-quadruplexes. G-quadruplex can form on a single DNA strand (left), or between two (middle) or four (right) DNA strands.

G4 DNA can arise in strands of guanine-rich DNA when guanine residues form Hoogsteen base parings to form a planar structure consisting of four guanines, otherwise known as a G-quartet (Figure 1A). These G-quartets can stack into G4 structures (Figure 1B). Canonical G4 structures form at the highly specific DNA motif G_3+_N_1-7_G_3+_N_1-7_G_3+_N_1-7_G_3+_, consisting of four runs of three or more guanines interspersed with variable length spacers containing any nucleotide. While canonical G4 DNA folds from a single DNA strand, G4 DNA can also form between two or even four separate strands of DNA [1]. More recently, the definition of G4-forming motifs has expanded to include those containing runs of two guanines, as well as spacers containing (many) more than seven nucleotides [2,3]. Given the high stability and wide range of potential G4 structures, is seems probable that mammalian cells have evolved different helicases with affinity for binding and unwinding of different G4 DNA structures. Interestingly, such helicases appear to have non-redundant functions in maintaining (epi-) genetic stability, as shown by the variable phenotypes caused by mutations in the associated genes. Here, we will discuss how we encountered three helicases for which we found evidence that one of their functions is to act upon G4 DNA structures in vivo. The strikingly different functions of these different helicase proteins illustrate the importance of proper G4 metabolism in maintaining genome stability. 

## 2. From Self-Renewal of Stem Cells to Telomeres

**Figure 2:**
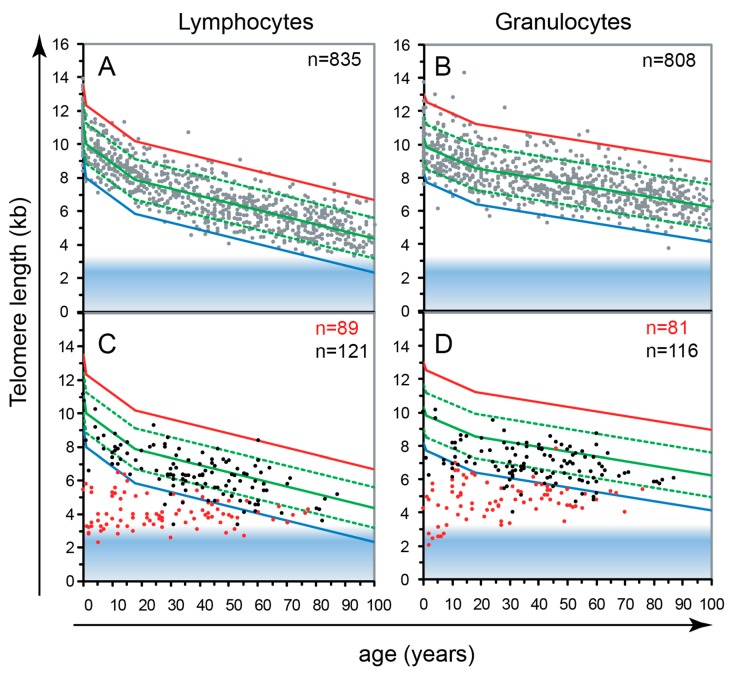
(**A**,**B**) Telomeres in human lymphocytes and granulocytes from peripheral blood shorten with age [15]. Fluorescence in situ hybridization followed by flow cytometry (Flow FISH) results from over 800 healthy individuals were used to calculate the distribution of telomere length at any given age (percentiles in population: solid green 50th; red 1st, blue 99th). Note the marked variation on average, cell specific telomere length at any given age. (**C**,**D**) The critical role of telomerase is illustrated by the telomere length in cells from patients that are haplo-insufficient for telomerase genes (red dots in C and D) compared to their unaffected siblings (black dots in C and D). The blue zone at the bottom of the graphs represents the area where telomeres are expected to be fully “uncapped” with less than 1 kb of TTAGGG repeats per chromosome end. Reproduced with permission from [12].

One prevalent idea in the late 1980s was that blood forming or hematopoietic stem cells (HSC) must be endowed with self-renewal properties in order to ensure blood cell formation over a lifetime. Transplantation studies in the mouse supported this notion in that single marked cells were shown to be capable of reconstituting blood cell formation in lethally irradiated recipients [4,5,6]. The situation in humans was less clear, but the assumption was that functional properties of human and murine HSCs would be comparable. However, we found that self-renewal properties of purified human HSC are developmentally controlled [7] and coincide with loss of telomere repeats with each division *in vitro* and *in vivo* [8]. Based on these observations, our studies shifted towards the role of telomeres in human biology [9] and we developed novel techniques using peptide nucleic acid probes to measure the length of telomere repeats in individual chromosomes [10] and single cells [11]. These techniques were used to show that the rate of telomere loss in human cells varies markedly between cell types and between individuals (Figure 2A,B). Later studies showed that telomerase RNA as well as the telomerase reverse transcriptase protein levels are both limiting stem cell function as a modest drop in those levels, resulting from haplo-insufficiency for either of these two telomerase genes, was found to have a dramatic effect on telomere length and stem cell function, often resulting in bone marrow failure or pulmonary fibrosis (Figure 2C,D from [12]). So in contrast to the mouse, where a single blood forming stem cell without telomerase can restore blood cell production in irradiated recipients [13], telomerase levels in human stem cells are very tightly controlled. Most likely, progressive telomere loss limits human stem cell proliferation to act as a tumor suppressor mechanism that does not exist in short-lived mice [14]. 

## 3. Hunting for Genes that Regulate Telomere Length

In view of the telomere loss in human cells, we became interested in factors, other than telomerase levels that could possibly explain the difference in the average telomere length in cells from individuals of the same age (Figure 2A,B). To study genetic control of telomere length we collaborated with Richard Hodes and others looking for genes that regulate telomere length in the mouse [16]. For this work, laboratory mice (*Mus musculus*) with very long tracks of telomere repeats (>30 kb) were crossed with a different murine species, *Mus spretus*, which has an average telomere length of around 10 kb. The F1 offspring of such crosses showed clear elongation of telomeres on *M.spretus* derived chromosomes. The F1 animals were backcrossed with *M. spretus* to map the *M.musculus* derived genetic loci required for this telomere elongation. Several loci were identified and we focused on a region of around 10 Mb at the tip of mouse chromosome 2 [16]. Because the mouse genome sequence had not been reported at the time, we studied the syntenic region on human chromosome 20 q. Several candidate genes, including “Novel Helicase Like (NHL)”, a gene with the seven conserved structural motifs of helicases and homology to yeast Rad3 were identified. Rather than trying to knock-out this “candidate” telomere length regulating gene in the mouse, we decided to study a homologous gene in *C.elegans*, a more suitable model organism for genetic studies [17].

## 4. Discovery of *dog-1*

**Figure 3:**
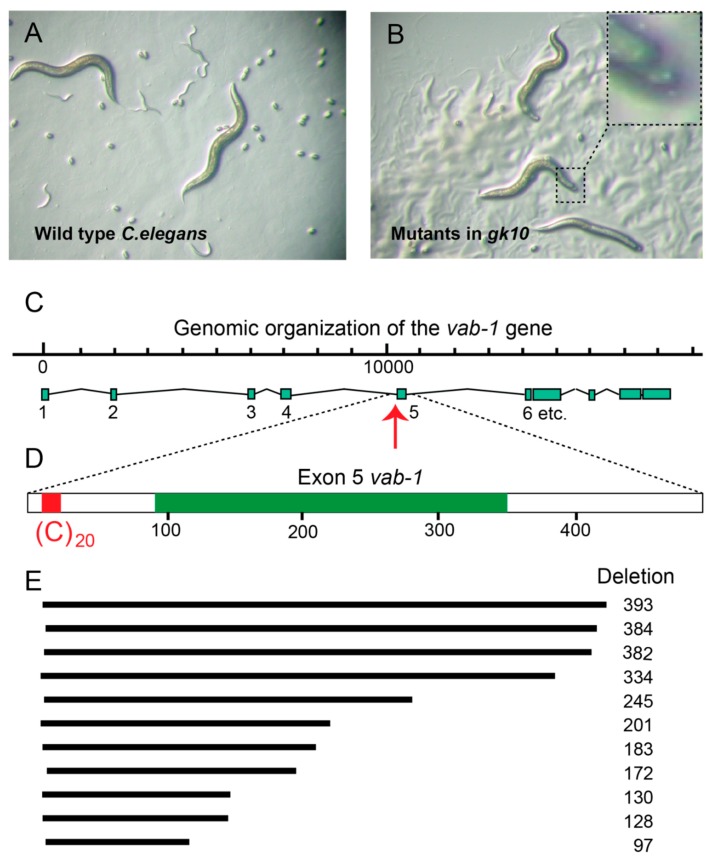
Deletions in helicase deficient *C.elegans* strain *gk10* show characteristic deletions throughout their genome that invariable start at the 3′ end of G-rich DNA. (**A**) Wild type animals. (**B**) Offspring of *gk10* with a “*variable abnormal* (*vab*)” notched head phenotype (insert). (**C**–**E**) Genetic complementation assays pointed to mutations in the *vab-1* gene and PCR studies revealed that the *vab* phenotype in *gk10* animals result from deletions close to exon 5 of the *vab-1* gene. Deletions that invariably start at the 3′ end of G-rich DNA were identified throughout the *C.elegans* genome in ~50% of poly guanine tracts longer than 18 G’s. Numbers represent base pairs.

A BLAST search of the human “NHL” gene against the *C.elegans* genome yielded several hits including F25H2.13 (now known as *rtel-1*) and F33H2.1, the second best hit. In collaboration with Ann Rose at UBC we studied F33H2.1 using a mutant strain (*gk10*) that lacked expression of the F33H2.1 protein [18]. The crucial finding was that animals with a characteristic “*Variable abnormal*” or *Vab* phenotype [19] were observed more frequently than predicted by chance in *gk10* (Figure 3B). *Gk10* animals with a *Vab* phenotype were crossed with known *Vab* mutants. Such complementation studies revealed that the phenotype in our helicase mutants resulted from loss of the *vab-1* gene. Strikingly, all *gk10* animals with a *Vab* phenotype had deletions in the *vab-1* gene initiating in front of exon 5 of (Figure 3C–E). Similar deletions were found in many other G-rich genomic regions in *gk10* and we decided to call the gene *Deletion of guanine-rich DNA* or *dog-1* assuming that more than one gene would be required to prevent the characteristic deletions in *gk10*. Marcel Tijsterman and colleagues set up a mutagenesis screen to look for such additional genes [20]. Using an elegant reporter strain, multiple independent mutants were indeed identified. Surprisingly, all these mutants were found to map to *dog-1*, supporting that DOG-1, now also known as FANCJ, is the lone helicase required for prevention of G-rich DNA deletions in nematodes. *Dog* would have been a better name! 

### 4.1. DOG-1/FANCJ Promotes Replication through G4 DNA

While *dog-1* mutant *C. elegans* displayed characteristic deletions of G-rich DNA, the animals did not appear to have telomere defects [18] even though our original goal was to identify factors involved in telomere length regulation. While we clearly did not find the NHL homolog in worms, DOG-1 must somehow be related to this mysterious factor. What could be the link between deletions in G-rich DNA and the setting of telomere length? In hindsight, it seems obvious that the answer is related to G4 DNA! Mammalian telomeres are made up of tandem TTAGGG repeats (TTAGGC in *C. elegans*) and readily form G4 structures in vitro [21]. Further investigation of the deletions in *dog-1* deficient worms showed that they coincide with the presence of G4 motifs at much higher rates than anywhere in the genome [20]. Deletions in *dog-1* mutant animals initiate at the 3′ end of G4 motifs and typically measure 100–200 nucleotides in length, indicating that G4 structures might form a replication barrier in the absence of *dog-1* [18,20]. Later it was shown that the human homolog of DOG-1, FANCJ (also called BACH1 or BRIP1), can bind and unwind G4 structures in vitro, and that FANCJ depletion sensitizes cells to G4 stabilizing agents [22]. Mutations in *FANCJ* are associated with Fanconi anemia, a genetic cancer-susceptibility disorder [23,24,25]. As in *C. elegans*, loss of function of FANCJ leads to genomic deletions in the vicinity of G4 motifs [26]. Finally, it was shown that FANCJ promotes replication fork progression through sites of G4 sequences [27,28], indicating that deletions occur due to replication fork stalling at G4’s, although the exact mechanism is still unknown. It should be noted that FANCJ plays other roles than maintaining genomic stability during DNA replication. FANCJ also promotes repair of DNA double strand breaks (DSBs) via the homologous recombination (HR) pathway through its interaction with BRCA1 [23,29]. For an excellent review of the many roles of FANCJ in maintaining genome stability the reader is referred elsewhere [30].

### 4.2. FANCJ Maintains Epigenetic Stability at G4 Motifs

**Figure 4:**
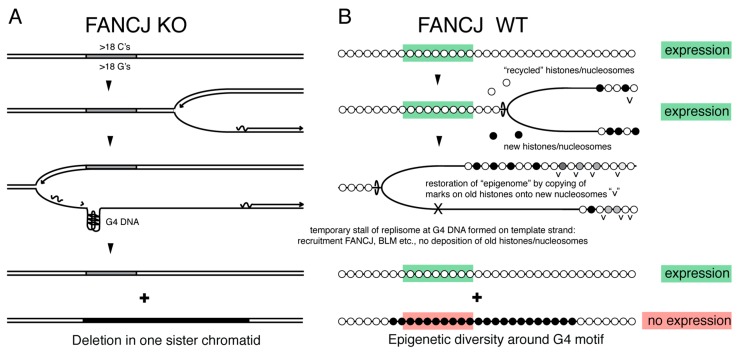
The silent sister hypothesis. (**A**) *C.elegans* lacking the DOG-1/FANCJ helicase show deletions throughout their genome that invariably start at the 3’ end of guanine tracts that are longer than 18 nucleotides [18]. (**B**) G4 DNA structures in normal cells, resolved by helicases such as FANCJ, RTEL, BLM and others, could drive epigenetic differences between sister chromatids as parental nucleosomes are unlikely to be still around for deposition onto nascent DNA by the time replication resumes. The “silent sister” hypothesis predicts that differences in gene expression between daughter cells can result at G4 locations from differences in the replication timing of G-rich DNA.

Despite the fact that G4 motifs can lead to deletions throughout the genome, these tracts are conserved throughout evolution. In fact, sequences with quadruplex forming potential are present at much higher than expected frequencies in most genomes, including in worms [31] and humans [32]. Why would evolution maintain these potentially pathogenic sequences if they did not serve a function? Although there is much speculation about what these functions are, it seems clear that G4 motifs in DNA can act as a switch to affect the transcription of nearby genes depending on whether a G4 structure is formed or not. Indeed, loss of FANCJ can cause epigenetic instability at genes containing G4 motifs, leading to both increases and decreases in gene expression in the absence of deletions [33]. While the presence of absence of a G4 can a directly affect transcriptional activity, FANCJ deficiency has also been linked to disruption of chromatin structure due to defects in restoring chromatin state behind replication forks [27]. FANCJ appears to maintain stability at G4 motifs in DNA in collaboration with two other G4 helicases, WRN and BLM [33,34]. Differences in the timing of sister chromatid replication caused by G4 DNA structures could trigger epigenetic differences between daughter cells after cell division, where one daughter inherits the “correct” chromatin state, while the other differs from the mother cell in gene expression at this locus (Figure 4). While such events would be more common in absence of G4 helicases or upon replications stress [35], local DNA replication timing differences could occur at low frequency in all dividing cells and impact gene expression on paired daughter cells as predicted by the “silent sister” hypothesis [36]. 

## 5. Discovery of RTEL1

**Figure 5:**
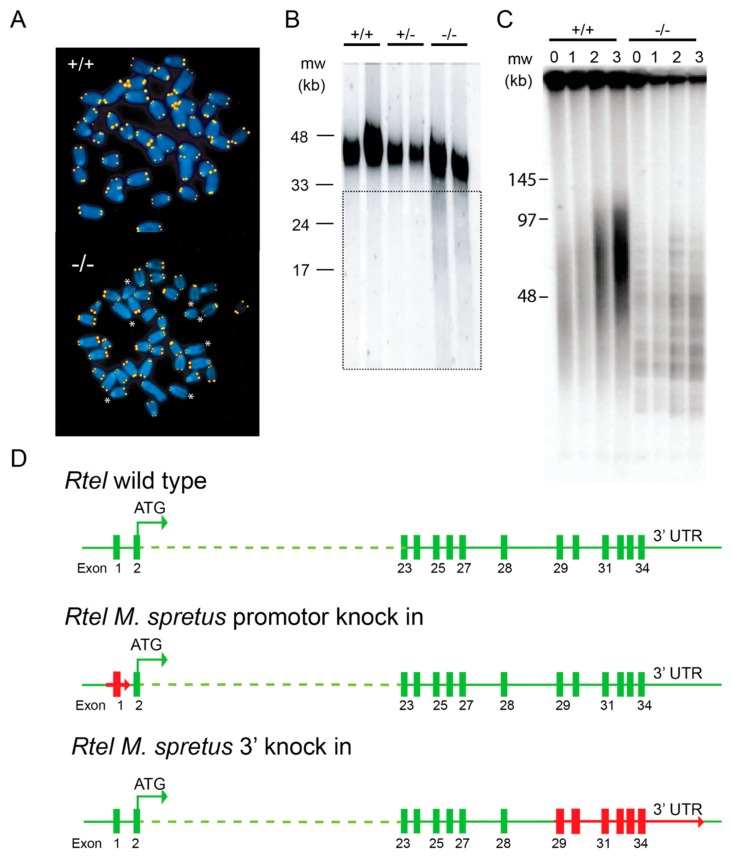
(**A**–**C**) RTEL is required for telomerase mediated extension of long telomeres. (**A**) Metaphase spreads of murine embryonic stem (ES) cells with (^+/+^) and without (^−/−^) *Rtel* were hybridized with fluorescently labeled (CCCTAA)_3_ peptide nucleic acid probes. Whereas all telomeres in *Rtel*^+/+^ chromosomes are labeled, fluorescence signals are either faint or missing on several *Rtel*^−/−^ chromosomes (asterisks). This result is in agreement with telomere restriction fragment (TRF) analysis (**B**) showing variable shortening of *Rtel*^−/−^ telomeres (boxed area). (**C**) High-resolution TRF analysis of ES cells with (left 4 lanes) and without *Rtel* (remaining lanes) shows a smear in wildtype cells and progressive shortening of discrete bands in cells lacking *Rtel* (for details see [39]). (**D**) To explore differences between *Rtel* in *M.musculus* and *M.spretus* that could explain the marked difference in telomere length between the two species we engineered knock-in animals using recombineering. For the first animal around 1 kb of the *M.spretus* promoter sequence flanking the untranslated exon 1 was inserted into the *M.musculus* genome of embryonic stem cells. Following selection and removal of the selectable marker a single loxP site (red arrow) was left next to the selected *M.spretus* sequence. A similar approach was used to replace 2.8 kb of *M.musculus* DNA including exons 29–34 at the 3′ end of the gene together with the UTR with corresponding sequences of *M. spretus*. Following injection of blastocyst heterozygous animals were obtained that were used to make homozygous knock-in animals as well as double knock-in animals. None of the knock-in animals showed a telomere phenotype.

Encouraged by the finding of DOG-1, a G4 DNA helicase in *C.elegans* and the known folding of telomeric DNA into G4 DNA [37], we returned to our search for the elusive NHL gene in the mammalian system. We cloned the gene and with help from Andras Nagy and Hao Ding we knocked out the “*NHL*” gene in the mouse. Animals without *NHL* died in utero at approximately E11, while developmental defects could already be detected at E8.5 [38]. However, we could obtain embryonic stem (ES) cells with and without *NHL*, the latter of which showed a clear telomere defect (Figure 5A–C). When *NHL*^+/−^ animals were crossed with *M.spretus*, we could show that *NHL* is indeed the gene required for elongation of *M.spretus* telomeres in crosses with *M.musculus*. We named the gene *Regulator of telomere length or Rtel*. This name was changed to *Rtel1* by the mouse nomenclature committee, no doubt assuming that more ***Rtel*** genes were to be discovered, a mistake we are familiar with.

### 5.1. RTEL1 Maintains Genome Stability and Telomere Integrity

*Rtel1^−/−^* ES cells grow slower and display shorter telomeres than ES cells derived from wildtype littermates (Figure 5B–D). Furthermore, *Rtel1^−/−^* ES cells show a severe differentiation defect that coincides with loss of telomere DNA and chromosomal rearrangements [38]. Since its discovery, *Rtel1* has been studied extensively by us and others. Shortly after our discovery of *Rtel1* in mice, the Boulton lab identified its homolog in *C. elegans*, naming it *rtel-1* [40]. Although *rtel-1* is not essential in worms, mutant animals do display increased lethality and germline apoptosis. Unlike *dog-1* mutants, *rtel-1* deficient worms do not display a deletion phenotype at G4 motifs, indicating that both proteins play different roles in maintaining genome stability. Deletion of *rtel-1* leads to increased sensitivity to DNA damaging agents and promotes DNA repair [40]. In mammalian cells, RTEL1 is required for normal DNA replication genome-wide, as well as telomere extension during DNA replication [39,40,41,42]. The latter function was linked to RTEL1 unwinding both T-loops and G4 structures in telomeres [41]. Specifically, RTEL1 deficiency leads to loss of terminal single stranded G-overhangs [43], known to preferentially form G4 structures [44]. Whether or not genome-wide DNA damage in absence of RTEL1 mainly occurs at G4 motifs as well is currently unknown. Mutations in the human *RTEL1* gene are now known to cause a particularly serious form of dyskeratosis congenita, the so-called Hoyeraal–Hreidarsson syndrome (HHS) [45]. Consistent with *Rtel1^−/−^* ES cells, cells from HHS patient show general genome instability with unusually short telomeres as well as increased telomere shortening compared to control cells.

### 5.2. RTEL1 and Telomerase

The RTEL1 requirement for elongation of short telomeres by telomerase was also documented using high-resolution TRF analysis [39]. Whereas wildtype embryonic stem (ES) cells show telomeres of variable length indicated by a smear in the TRF analysis, RTEL1 deficient ES cells show progressive shortening of telomeres in multiple discrete bands (Figure 5C). We interpreted these finding as support for the notion that RTEL1 is required for elongation of terminal telomeric DNA by telomerase. This raises many questions about telomeres in *M.spretus*. Does telomerase in *M.spretus* only act on very short telomeres [46]? Does single stranded G-rich DNA at the 3’ end of *M.spretus* chromosomes fold into a G4 structure that does not exist in *M.musculus*? Do G4 structures at the very 3’ end of chromosomes exist in cells from other species? How are such structures hidden from DNA damage response pathways? Do terminal G4 DNA structures compete with T-loops [47] or do T-loops themselves contain G4 structures? Does terminal G4 DNA explain why G4 motifs are common in telomere DNA from almost all organisms with linear chromosomes? Clearly, much more work needs to be done to elucidate the roles of G4 DNA, RTEL1 and telomerase in relation to telomere function in different cells from different species. A recent piece to the puzzle was the finding that reversed replication forks are a pathological substrate for telomerase and a source of telomere catastrophe in *Rtel1*^−/−^ cells [48]. Since telomeres harbor the highest density of G4 motifs in the entire genome, compromised telomere integrity in the absence of the G4 DNA helicase RTEL1 is not unexpected. 

### 5.3. What is Wrong with Rtel1 in M.spretus?

Once *Rtel1* had been identified as a gene required for telomere elongation, our studies focused on the difference between *Rtel1* in *M.musculus* and *M.spretus*. Given the highly conserved predicted amino acid sequence between the *Mus* species, we focused on differences in the promotor region, where one G4 DNA motif is lost in *M.spretus*, and the 3′ end of the gene, in view of the noticeable differences in the splicing of 3′ exons between the species [38]. For these studies the promotor region as well as the 3’ end of the *Rtel1* gene in *M.musculus* were replaced with sequences from *M.spretus* (Figure 5D). This was a herculean effort by Evert-Jan Uringa in the lab using recombineering [49] at a time when CRISPR-Cas9 mediated gene editing had not yet been invented. Evert-Jan successfully made two knock-in strains, replacing the promotor as well as the 3′ end of *Rtel1* in *M.musculus* with sequences from *M.spretus* as shown in Figure 5D. Unfortunately, neither of the knock-in animals showed a telomere phenotype. Even the double knock-in animals, obtained upon crossing the knock-in animals, had telomeres that were indistinguishable in length from wild type animals (results not shown). On hindsight we should probably have focused on the methionine at position 482 in RTEL1 from *M.musculus* with the lysine observed at that position in *M.spretus* [38]. More recent studies have shown that telomere dysfunction in cells from HHS patients can result from a M482I mutation in RTEL1 [45]. The methionine at position 482 in RTEL was furthermore recently reported to be within the Arch domain required for DNA binding and translocation [50]. Whether the M482K amino acid substitution in RTEL1 indeed explains the telomere length difference between *M.musculus* and *M.spretus* remains to be shown. 

## 6. BLM is A Multifunctional Caretaker of Genome Stability

DOG-1 and FANCJ protect against deletions at G4 motifs, and FANCJ maintains epigenetic stability in collaboration with the BLM helicase. BLM is one of the best studied G4 helicases, although its role in maintaining (epi-) genetic stability is not fully understood. BLM was first identified as the causative factor in Bloom syndrome, a genetic disorder characterized by growth retardation, genetic instability, and cancer predisposition [51,52]. The main phenotype of BLM deficient cells includes sensitivity to a range of DNA damaging agents, elevated spontaneous mutation rates, and a nearly 10-fold increase is sister chromatid exchange (SCE) events [53]. Like FANCJ, BLM plays multiple roles in maintaining genome stability, and it is, therefore, challenging to separate BLM’s function in G4 biology from its other functions. The BLM helicase can unwind a wide range of DNA structures, including B-DNA [54], but it shows much higher affinity for double-Holliday junctions [55] and G4 DNA [56,57]. It was shown that BLM is an anti-recombinase that prevents exchanges of genetic material between sister chromatids and homologs during homologous recombination [58]. Further roles where discovered in maintaining genome stability at replication forks [59,60,61], resolving chromosome bridges during mitosis [62,63], and telomere maintenance [64]. We will not attempt to summarize the extensive literature on the different functions of BLM here and readers are referred to the primary literature and the many excellent reviews written on the subject. Instead, we focus on BLM’s role in processing of G4 DNA.

### 6.1. BLM Promotes Telomere Replication

G4 DNA structures readily form in telomeric DNA and these need to be processed for proper telomere replication. BLM localizes to telomeres [64,65] and interacts with several components of the shelterin complex [66]. BLM activity was found to be enhanced by two of these components, TRF2 [67] and POT1 [68], perhaps allowing BLM to unwind or assist in the unwinding of telomeric G4 DNA during replication. Indeed, BLM deficiency leads to a decrease in the speed of replication at telomeres, but only in the G-rich strand which is capable of folding into G-quadruplexes [69]. This suggests that BLM is specifically required to unfold G4 structures during telomere replication. Indeed, telomere replication can also be retarded by treating cells with G4 stabilizers [69], and the absence of BLM leads to increased telomere fragility and telomere shortening [64].

### 6.2. BLM Prevents Replication fork Stalling and Recombination at G-Quadruplexes

**Figure 6:**
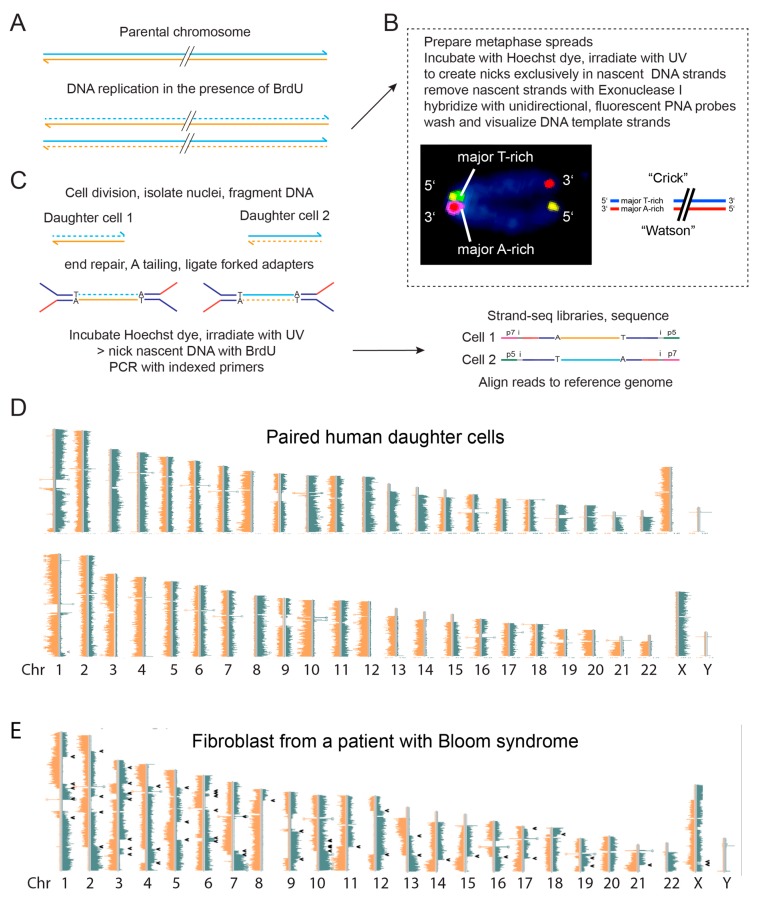
Principle of strand-specific DNA analysis. (**A**) Following one full round of replication in the presence of BrdU, only nascent DNA strands have incorporated BrdU (dotted lines). (**B**) If chromosomes are arrested at metaphase and spread onto a slide, it is possible to prepare completely single stranded chromosome spreads by nicking the DNA using Hoechst and UV followed by digestion with exonuclease. Single stranded chromosomes can be used to identify the 5′ and 3′ ends of chromosomes and study the orientation of genomic segments relative to such ends with unidirectional FISH probes. (**C**) If cells are allowed to divide after one round of BrdU, the two daughter cells will inherit one template strand from each parent. Such parental template strands can be identified using single cell Strand-seq as illustrated in (**D**) Reads derived from each parental chromosome map either the 5′ to 3′ “Crick” template strand or to the 3′ to 5′ “Watson” template strand. If during the synthesis of parental DNA template strands were switched reads will map to opposite sides of the reference genome at the site of such an exchange (arrows). Note the single chromatid exchange event in the paired normal cells (bottom of chr 1) and the many template strand exchanges in cells from a patient with Bloom’s syndrome (**E**) The latter are not randomly distributed over the genome but enriched at G4 motifs of active genes [77].

Since BLM is required to replicate through G4 structures in telomeres, is the same true for other locations in the genome? We became curious if SCE locations in BLM deficient cells overlapped with G4 motifs. There is evidence that the presence of a stabilized G4 structure delays BLM in unwinding duplex DNA [70], but classical cytogenetic SCE identification methods do not allow for high-resolution mapping of these events. To improve on this, we used Strand-seq, a sequencing-based method to map SCEs at kilobase resolution in single cells [71,72]. We previously showed that it was possible to distinguish between parental DNA strands and newly synthesized DNA in newly replicated cells by using unidirectional fluorescence in situ hybridization (FISH) probes after one round of incorporation with bromodeoxyuridine BrdU [73]. The approach we took, shown in Figure 6, is based on the nicking of nascent DNA with BrdU following exposure of the DNA to UV light in the presence of the DNA dye Hoechst 33258 [74]. Normally, the Hoechst dye binds with high affinity to double stranded DNA and shows fluorescence upon excitation with UV light. Such fluorescence is not observed when Hoechst is bound to BrdU substituted DNA. In this case, the absorbed light energy is not emitted but initiates a photochemical reaction resulting in nick exclusively in the BrdU substituted DNA strand (Figure 6). This principle [75] was exploited to show that major satellite sequences in murine chromosomes are always oriented in the same direction relative to the 3’ end of G-rich telomeric DNA (Figure 6B) [73]. We adopted this method into a sequencing-based approach, allowed identification and mapping of SCE events at kilobase resolution [71,72]. Using the Strand-seq method we confirmed that BLM deficient cells have elevated levels of SCEs [76,77]. Furthermore, we could show that SCEs in BLM deficient cells are not randomly distributed over the genome but are enriched at genes in general and genes with G4 motifs in particular [77]. Interestingly, this effect was strongest for G4 motifs in the strands of transcribed genes, highlighting the interplay between transcription and G4 formation. 

### 6.3. BLM Deficiency Affects Transcription of Genes Containing G4 Motifs

As discussed above, BLM appears to cooperate with FANCJ to impact epigenetic stability, at least in part by assuring proper recycling of parental histones behind the replication forks [33,34]. There have been no reports of epigenetic instability in the absence of BLM alone, although changes in transcriptional profiles were reported [78,79]. These changes in gene expression occur in both directions, and correlate with the presence of G4 motifs in both promoters and gene bodies. There is also evidence that BLM prevents transcription-induced DNA damage, specifically by unwinding R-loops that occur during transcription [80,81]. While R-loops are not necessarily associated with G4s, there are indications that R-loops form more readily and are more stable when a G4 motif is present on the non-transcribed strand, perhaps by stabilizing the displaced DNA strand [82]. BLM has some affinity for R-loops, similar to D-loops, but it is also possible that BLM destabilizes R-loops by unwinding the G4 on the displaced DNA strand.

## 7. Molecular Phenotypes That Inform *in vivo* Helicase Function

In the case of DOG-1 or FANCJ we were fortunate to stumble upon a very specific, molecular “signature” phenotype observed in mutant cells (Figure 3C–E). The ability to deduce functional properties of proteins acting on DNA by analysis of mutant cells is also illustrated for RTEL1 in Figure 5C and for BLM in Figure 6E. This type of information is vital to complement structural information about proteins which is proceeding at an unprecedented pace [83]. In case of the iron-sulfur cluster (Fe-S) helicases DOG-1, FANCJ, and RTEL1, the generation of structural information has been relatively slow. Specific proteins and mitochondria are required for biosynthesis and incorporation of the FeS cluster into such proteins [84,85,86] and such conditions are not easily reproduced in vitro. The functional role of the FeS cluster is furthermore far from clear. It has been suggested that next to a structural role in protein folding and a functional role in 5′ to 3′ translocation on DNA substrates [50,87], the FeS cluster could also increase the binding affinity for non-duplex DNA structures [88]. G4 DNA has furthermore been reported to have peroxidase activity upon interaction with heme inside the nucleus [89] and perhaps G4 structures and G4 helicases communicate in a language that we still need to learn. The answers to the many questions about G4 DNA and G4 DNA helicases will no doubt require the development of novel tools and novel insight. Sydney Brenner once quipped “Progress in science depends on new techniques, new discoveries and new ideas, probably in that order” [90]. New techniques and discoveries enabled by such techniques are needed to help solve some of the current riddles related to G4 DNA and G4 helicases. 

## 8. Conclusions

DNA with a G4 motif can function as a molecular switch by conversion of G4 DNA into duplex DNA and vice versa. Where such switches are useful to cells and organisms remains to be fully understood. However, switches enabled by G4 motifs are a double-edged sword: during replication and recombination G4 structures are obstacles that need to be resolved. As such, the presence of G4 structures at any given genomic site needs to be closely controlled. We have discussed three different helicases which while all capable of acting upon G4 DNA, play highly distinct roles in protecting against G4-associated damage. Many other G4 DNA helicases have been identified, with both unique and overlapping functions. For example, both yeast and human PIF1 proteins are potent G4 helicases [91,92]. Yeast Pif1 promotes replication through G4 motifs [93,94], and its absence destabilizes such motifs [93,95]. These results are reminiscent of DOG-1/FANCJ, and it is unclear how much functional overlap there is between these different G4 helicases. Other known G4 helicases include ATRX [96], WRN [97,98], the BLM and WRN homolog in S. cerevisiae, Sgs1 [99], XPB and XPD [100] and many others. What is the basis for the strikingly different cellular functions and phenotypes of loss of these different G4 helicases? Are these differences caused ‘simply’ by different spatiotemporal recruitment to G4 DNA, or do these helicases only bind and unwind certain classes of G4 structures in vivo [101]? With current genome editing and some of the tools described in this paper the answer to these and related questions should be forthcoming in the not so distant future.
